# Supplementary material for: Using virtual reality and thermal imagery to improve statistical modelling of vulnerable and protected species
Source: PLoS One. 2019 Dec 11;14(12):e0217809. doi: 10.1371/journal.pone.0217809 (PMC6905580; doi:10.1371/journal.pone.0217809)
Supplement: S1 Table — Koala presence/absence and habitat data collected during field surveys of the study area or derived from freely available geographic information system (GIS) datasets. (DOCX) [file pone.0217809.s001.docx]

Supporting information for Leigh et al. “Using virtual reality and thermal imagery to improve statistical modelling of vulnerable and protected species” published by PLoS ONE.

**S1 Table. Koala and habitat data.** Koala presence/absence and habitat data collected during field surveys of the study area or derived from freely available geographic information system (GIS) datasets.

| **Site code** | **Latitude** | **Longitude** | **Date*** | **Survey type*** | **Source*** | **Koala P/A (1/0)** | **Weight (0 to 1)** | **FPC (%)** | **REV (0/1)** | **Path (m)** | **Water (m)** |
| --- | --- | --- | --- | --- | --- | --- | --- | --- | --- | --- | --- |
| W14 | -27.7014 | 153.1871 | 19/11/2012 | Ground | LCC | 1 | 1 | 36 | 1 | 10.55 | 12.16 |
| W15 | -27.7007 | 153.1884 | 17/11/2013 | Ground | LCC | 1 | 1 | 40 | 0 | 123.76 | 73.93 |
| W16 | -27.7021 | 153.1881 | 12/01/2014 | Ground | LCC | 1 | 1 | 68 | 1 | 4.20 | 17.72 |
| W17 | -27.7006 | 153.1892 | 8/04/2014 | Ground | LCC | 1 | 1 | 58 | 0 | 192.65 | 33.88 |
| W19 | -27.7013 | 153.19 | 13/08/2014 | Ground | LCC | 1 | 1 | 39 | 0 | 114.38 | 34.45 |
| W20 | -27.7025 | 153.1888 | 23/10/2014 | Ground | LCC | 1 | 1 | 66 | 1 | 0.64 | 19.27 |
| W21 | -27.7008 | 153.1924 | 7/08/2016 | Ground | LCC | 1 | 1 | 51 | 1 | 25.72 | 8.88 |
| W22 | -27.7011 | 153.1923 | 12/08/2016 | Ground | LCC | 1 | 1 | 59 | 0 | 8.35 | 18.18 |
| W23 | -27.7012 | 153.1917 | 12/08/2016 | Ground | LCC | 1 | 1 | 48 | 1 | 2.54 | 11.16 |
| W24 | -27.7019 | 153.1878 | 24/09/2016 | Ground | LCC | 1 | 1 | 69 | 1 | 6.31 | 14.28 |
| W25 | -27.7007 | 153.1911 | 30/09/2016 | Ground | LCC | 1 | 1 | 53 | 0 | 37.41 | 42.15 |
| W26 | -27.7017 | 153.1883 | 5/10/2016 | Ground | LCC | 1 | 1 | 24 | 0 | 46.55 | 30.88 |
| W27 | -27.7023 | 153.1892 | 5/10/2016 | Ground | LCC | 1 | 1 | 66 | 1 | 32.21 | 16.54 |
| W34 | -27.701 | 153.192 | 16/10/2016 | Ground | LCC | 1 | 1 | 60 | 0 | 16.93 | 9.24 |
| W40 | -27.7013 | 153.1919 | 17/01/2017 | Ground | LCC | 1 | 1 | 44 | 1 | 10.06 | 10.48 |
| W41 | -27.7019 | 153.1879 | 19/12/2017 | Ground | LCC | 1 | 1 | 68 | 1 | 1.61 | 8.54 |
| W56 | -27.702 | 153.188 | 19/12/2017 | Ground | This study | 1 | 1 | 68 | 1 | 2.91 | 12.40 |
| W1 | -27.7011 | 153.1915 | 4/10/2016 | Thermal | This study | 1 | 1 | 50 | 1 | 20.66 | 8.68 |
| W10 | -27.7027 | 153.1922 | 20/10/2016 | Thermal | This study | 1 | 1 | 55 | 1 | 37.53 | 62.44 |
| W12 | -27.6994 | 153.1902 | 20/10/2016 | Thermal | This study | 1 | 0.5 | 57 | 0 | 140.05 | 12.89 |
| W2 | -27.7024 | 153.1932 | 4/10/2016 | Thermal | This study | 1 | 1 | 26 | 0 | 65.65 | 3.03 |
| W3 | -27.7023 | 153.1919 | 4/10/2016 | Thermal | This study | 1 | 1 | 58 | 1 | 37.00 | 25.44 |
| W4 | -27.7021 | 153.1914 | 4/10/2016 | Thermal | This study | 1 | 1 | 58 | 1 | 29.66 | 5.25 |
| W43 | -27.7013 | 153.1907 | 19/12/2017 | Thermal | This study | 1 | 0.9 | 50 | 0 | 72.52 | 25.71 |
| W44 | -27.7021 | 153.1929 | 19/12/2017 | Thermal | This study | 1 | 0.5 | 51 | 1 | 56.50 | 19.74 |
| W45 | -27.7021 | 153.1882 | 19/12/2017 | Thermal | This study | 1 | 0.9 | 68 | 1 | 12.12 | 13.17 |
| W46 | -27.7024 | 153.1893 | 20/12/2017 | Thermal | This study | 1 | 0.5 | 66 | 1 | 23.74 | 23.47 |
| W47 | -27.7016 | 153.1888 | 20/12/2017 | Thermal | This study | 1 | 0.5 | 25 | 0 | 92.77 | 46.35 |
| W48 | -27.703 | 153.1913 | 20/12/2017 | Thermal | This study | 1 | 0.9 | 59 | 1 | 14.21 | 46.84 |
| W49 | -27.7022 | 153.1923 | 20/12/2017 | Thermal | This study | 1 | 0.5 | 57 | 1 | 2.08 | 6.67 |
| W5 | -27.7018 | 153.1906 | 4/10/2016 | Thermal | This study | 1 | 1 | 65 | 1 | 33.34 | 19.40 |
| W50 | -27.7022 | 153.1905 | 20/12/2017 | Thermal | This study | 1 | 0.9 | 45 | 1 | 8.82 | 0.84 |
| W51 | -27.7027 | 153.1907 | 20/12/2017 | Thermal | This study | 1 | 0.9 | 70 | 1 | 21.11 | 34.54 |
| W52 | -27.7032 | 153.1923 | 20/12/2017 | Thermal | This study | 1 | 0.9 | 59 | 1 | 4.55 | 31.54 |
| W53 | -27.7015 | 153.19 | 20/12/2017 | Thermal | This study | 1 | 0.9 | 39 | 0 | 99.80 | 17.87 |
| W54 | -27.703 | 153.1919 | 20/12/2017 | Thermal | This study | 1 | 0.5 | 52 | 1 | 34.13 | 62.21 |
| W55 | -27.7025 | 153.193 | 20/12/2017 | Thermal | This study | 1 | 0.9 | 52 | 1 | 43.87 | 17.81 |
| W6 | -27.7027 | 153.1915 | 4/10/2016 | Thermal | This study | 1 | 1 | 49 | 1 | 50.38 | 70.40 |
| W7 | -27.7029 | 153.1909 | 20/10/2016 | Thermal | This study | 1 | 0.5 | 61 | 1 | 13.67 | 44.37 |
| W8 | -27.7028 | 153.1929 | 20/10/2016 | Thermal | This study | 1 | 1 | 52 | 1 | 22.44 | 27.67 |
| W9 | -27.7026 | 153.1934 | 20/10/2016 | Thermal | This study | 1 | 0.5 | 27 | 0 | 75.33 | 9.24 |
| SP01 | -27.7018 | 153.1899 | 18/12/2017 | Ground-Thermal | This study | 0 | 0.9 | 49 | 0 | 76.52 | 28.22 |
| SP02 | -27.7021 | 153.1887 | 18/12/2017 | Ground-Thermal | This study | 0 | 0.9 | 38 | 1 | 39.04 | 10.10 |
| SP04 | -27.6998 | 153.1896 | 18/12/2017 | Ground-Thermal | This study | 0 | 0.9 | 66 | 0 | 208.19 | 12.47 |
| SP06 | -27.702 | 153.1892 | 18/12/2017 | Ground-Thermal | This study | 0 | 0.9 | 59 | 0 | 64.14 | 16.44 |
| SP07 | -27.7016 | 153.192 | 18/12/2017 | Ground-Thermal | This study | 0 | 0.9 | 48 | 0 | 30.58 | 9.04 |
| SP09 | -27.7009 | 153.1875 | 18/12/2017 | Ground-Thermal | This study | 0 | 0.9 | 0 | 0 | 51.93 | 55.18 |
| SP10 | -27.7012 | 153.189 | 18/12/2017 | Ground-Thermal | This study | 0 | 0.9 | 28 | 0 | 135.12 | 92.80 |
| SP12 | -27.7022 | 153.1904 | 18/12/2017 | Ground-Thermal | This study | 0 | 0.9 | 61 | 0 | 12.83 | 6.70 |
| SP13 | -27.7024 | 153.1917 | 18/12/2017 | Ground-Thermal | This study | 0 | 0.9 | 58 | 1 | 58.77 | 33.61 |
| SP15 | -27.7001 | 153.1886 | 18/12/2017 | Ground-Thermal | This study | 0 | 0.9 | 41 | 0 | 183.74 | 18.89 |
| SP17 | -27.7006 | 153.1889 | 18/12/2017 | Ground-Thermal | This study | 0 | 0.9 | 0 | 0 | 171.33 | 39.22 |
| SP19 | -27.7027 | 153.1897 | 18/12/2017 | Ground-Thermal | This study | 0 | 0.9 | 67 | 1 | 0.13 | 28.99 |
| SP20 | -27.6996 | 153.1873 | 18/12/2017 | Ground-Thermal | This study | 0 | 0.9 | 40 | 0 | 132.81 | 56.33 |
| SP21 | -27.7008 | 153.1898 | 18/12/2017 | Ground-Thermal | This study | 0 | 0.9 | 66 | 0 | 152.30 | 19.29 |
| SP25 | -27.7008 | 153.1907 | 18/12/2017 | Ground-Thermal | This study | 0 | 0.9 | 55 | 0 | 65.08 | 41.29 |
| SP26 | -27.7011 | 153.1919 | 18/12/2017 | Ground-Thermal | This study | 0 | 0.9 | 60 | 0 | 8.99 | 9.83 |
| SP28 | -27.7017 | 153.1877 | 18/12/2017 | Ground-Thermal | This study | 0 | 0.9 | 0 | 1 | 5.48 | 1.97 |
| SP30 | -27.7018 | 153.1893 | 18/12/2017 | Ground-Thermal | This study | 0 | 0.9 | 73 | 0 | 88.31 | 39.97 |
| SP31 | -27.7005 | 153.1913 | 18/12/2017 | Ground-Thermal | This study | 0 | 0.9 | 59 | 0 | 47.78 | 22.69 |
| SP32 | -27.7028 | 153.1894 | 18/12/2017 | Ground-Thermal | This study | 0 | 0.9 | 70 | 1 | 17.41 | 5.27 |
| SP33 | -27.7017 | 153.1882 | 18/12/2017 | Ground-Thermal | This study | 0 | 0.9 | 0 | 0 | 38.63 | 26.33 |
| SP34 | -27.7013 | 153.191 | 18/12/2017 | Ground-Thermal | This study | 0 | 0.9 | 57 | 1 | 49.42 | 1.54 |
| SP35 | -27.7018 | 153.1876 | 18/12/2017 | Ground-Thermal | This study | 0 | 0.9 | 0 | 1 | 9.26 | 16.78 |
| SP37 | -27.7024 | 153.191 | 18/12/2017 | Ground-Thermal | This study | 0 | 0.9 | 57 | 1 | 39.91 | 30.42 |
| SP38 | -27.6999 | 153.1889 | 18/12/2017 | Ground-Thermal | This study | 0 | 0.9 | 59 | 0 | 219.98 | 17.47 |
| SP39 | -27.7001 | 153.1902 | 18/12/2017 | Ground-Thermal | This study | 0 | 0.9 | 49 | 0 | 147.32 | 5.47 |
| SP40 | -27.7005 | 153.1915 | 18/12/2017 | Ground-Thermal | This study | 0 | 0.9 | 46 | 0 | 39.78 | 2.98 |
| SP42 | -27.6998 | 153.1903 | 18/12/2017 | Ground-Thermal | This study | 0 | 0.9 | 56 | 0 | 164.58 | 9.60 |
| SP43 | -27.7022 | 153.1877 | 18/12/2017 | Ground-Thermal | This study | 0 | 0.9 | 0 | 1 | 34.10 | 7.43 |
| SP44 | -27.7021 | 153.1913 | 18/12/2017 | Ground-Thermal | This study | 0 | 0.9 | 58 | 1 | 26.15 | 3.46 |
| SP45 | -27.6995 | 153.189 | 18/12/2017 | Ground-Thermal | This study | 0 | 0.9 | 68 | 0 | 220.84 | 2.13 |
| SP47 | -27.7008 | 153.1905 | 18/12/2017 | Ground-Thermal | This study | 0 | 0.9 | 55 | 0 | 84.18 | 21.77 |
| SP48 | -27.7002 | 153.188 | 18/12/2017 | Ground-Thermal | This study | 0 | 0.9 | 71 | 0 | 139.72 | 43.23 |
| SP50 | -27.7029 | 153.1923 | 19/12/2017 | Ground-Thermal | This study | 0 | 0.9 | 52 | 1 | 29.20 | 64.77 |
| SP51 | -27.7031 | 153.1916 | 19/12/2017 | Ground-Thermal | This study | 0 | 0.9 | 56 | 1 | 11.07 | 45.86 |
| SP52 | -27.7032 | 153.1911 | 19/12/2017 | Ground-Thermal | This study | 0 | 0.9 | 60 | 1 | 11.56 | 19.01 |
| SP53 | -27.7034 | 153.1922 | 19/12/2017 | Ground-Thermal | This study | 0 | 0.9 | 59 | 1 | 16.65 | 11.71 |
| SP54 | -27.7013 | 153.1882 | 18/12/2017 | Ground-Thermal | This study | 0 | 0.9 | 9 | 0 | 69.86 | 62.98 |
| SP55 | -27.7022 | 153.1926 | 18/12/2017 | Ground-Thermal | This study | 0 | 0.9 | 61 | 1 | 27.14 | 11.88 |
| SP56 | -27.7014 | 153.1904 | 18/12/2017 | Ground-Thermal | This study | 0 | 0.9 | 0 | 0 | 78.75 | 14.76 |
| SP57 | -27.7032 | 153.1931 | 19/12/2017 | Ground-Thermal | This study | 0 | 0.9 | 57 | 0 | 55.51 | 7.23 |

* Pertains to koala survey data. Ground, ground surveys; Thermal, aerial thermal-imagery surveys; LCC, Logan City Council; P/A, presence/absence; Weight, confidence rating in koala P/A; FPC, foliage projective cover; REV, remnant *Eucalyptus* vegetation (0 = no; 1 = yes); Path, distance to nearest path; Water, distance to nearest fresh water.
