# Supplementary material for: Using virtual reality and thermal imagery to improve statistical modelling of vulnerable and protected species
Source: PLoS One. 2019 Dec 11;14(12):e0217809. doi: 10.1371/journal.pone.0217809 (PMC6905580; doi:10.1371/journal.pone.0217809)
Supplement: S2 Table — Parameter estimates for the fixed effects in the ground survey only (G), ground and thermal-imagery survey (GT), combined ground-survey and expert-elicitation (G_E), and combined ground and thermal-imagery survey and expert-elicitation (GT_E) models. (DOCX) [file pone.0217809.s002.docx]

Supporting information for Leigh et al. “Using virtual reality and thermal imagery to improve statistical modelling of vulnerable and protected species” published by PLoS ONE.

**S2 Table. Fixed effects tables.** Parameter estimates for the fixed effects in the ground survey only (G), ground and thermal-imagery survey (GT), combined ground-survey and expert-elicitation (G_E), and combined ground and thermal-imagery survey and expert-elicitation (GT_E) models.

| **Model** | **Covariate** | **Estimate** | **Standard error** |
| --- | --- | --- | --- |
| **G** | Intercept | 0.018 | 0.455 |
|  | FPC | 0.199 | 0.497 |
|  | REV | 0.736 | 0.732 |
|  | Water | 0.251 | 0.514 |
|  | Path | -1.378 | 0.727 |
|  | Latitude | 1.882 | 0.832 |
|  | Longitude | -0.599 | 0.590 |
| **GT** | Intercept | 0.018 | 0.277 |
|  | FPC | 0.266 | 0.245 |
|  | REV | 0.527 | 0.385 |
|  | Water | 0.291 | 0.240 |
|  | Path | -0.686 | 0.419 |
|  | Latitude | 0.447 | 0.393 |
|  | Longitude | 0.244 | 0.275 |
| **G_E** | Intercept | 0.087 | 0.394 |
|  | FPC | 0.128 | 0.148 |
|  | REV | 0.013 | 0.280 |
|  | Water | -0.486 | 0.133 |
|  | Path | -0.114 | 0.229 |
|  | Latitude | -0.259 | 0.292 |
|  | Longitude | 0.172 | 0.182 |
| **GT_E** | Intercept | 0.049 | 0.262 |
|  | FPC | 0.163 | 0.131 |
|  | REV | 0.133 | 0.238 |
|  | Water | -0.335 | 0.119 |
|  | Path | -0.152 | 0.209 |
|  | Latitude | -0.171 | 0.244 |
|  | Longitude | 0.250 | 0.157 |
