# Supplementary material for: Using virtual reality and thermal imagery to improve statistical modelling of vulnerable and protected species
Source: PLoS One. 2019 Dec 11;14(12):e0217809. doi: 10.1371/journal.pone.0217809 (PMC6905580; doi:10.1371/journal.pone.0217809)
Supplement: S3 Table — Virtual-reality elicited information from experts viewing 360-degree images of potential koala habitat. (DOCX) [file pone.0217809.s003.docx]

Supporting information for Leigh et al. “Using virtual reality and thermal imagery to improve statistical modelling of vulnerable and protected species” published by PLoS ONE.

**S3 Table. Expert elicitation data.** Virtual-reality elicited information from experts viewing 360-degree images of potential koala habitat. Question (Q) numbers as per S1 File.

| Site | Expert | Q3 | Q4 | Q5 | Q6 |
| --- | --- | --- | --- | --- | --- |
| W56 | 1 | 0.25 | 2 | 0.50 | 2 |
| W18 | 1 | 0 | 2 | 0.05 | 3 |
| W31 | 1 | 0.25 | 2 | 0.50 | 2 |
| W33 | 1 | 0.1 | 1 | 0.50 | 2 |
| W36 | 1 | 0.25 | 2 | 0.50 | 2 |
| SP04 | 1 | 0.05 | 1 | 0.30 | 2 |
| SP09 | 1 | 0 | 3 | 0 | 3 |
| SP13 | 1 | 0.1 | 2 | 0.40 | 2 |
| SP26 | 1 | 0.25 | 2 | 0.40 | 2 |
| SP51 | 1 | 0.25 | 2 | 0.50 | 2 |
| W26 | 2 | 0.4 | 1 | 1 | 3 |
| W28 | 2 | 0.4 | 1 | 1 | 2 |
| W29 | 2 | 0.8 | 1 | 1 | 3 |
| W39 | 2 | 0.8 | 1 | 0.80 | 3 |
| SP21 | 2 | 0.1 | 1 | 0.70 | 2 |
| SP43 | 2 | 0.2 | 1 | 0.60 | 2 |
| SP45 | 2 | 0.2 | 1 | 0.60 | 3 |
| SP47 | 2 | 0.5 | 1 | 1 | 3 |
| SP53 | 2 | 0.1 | 2 | 0.80 | 3 |
| SP56 | 2 | 0.5 | 1 | 1 | 3 |
| W56 | 3 | 0.7 | 2 | 0.9 | 3 |
| W18 | 3 | 0.2 | 2 | 0.3 | 2 |
| W31 | 3 | 0.8 | 2 | 0.90 | 3 |
| W33 | 3 | 0.7 | 2 | 0.90 | 2 |
| W36 | 3 | 0.7 | 2 | 0.70 | 2 |
| SP04 | 3 | 0.3 | 2 | 0.40 | 2 |
| SP09 | 3 | 0.3 | 2 | 0.20 | 3 |
| SP13 | 3 | 0.5 | 2 | 0.80 | 3 |
| SP26 | 3 | 0.7 | 2 | 0.70 | 2 |
| SP51 | 3 | 0.8 | 2 | 0.80 | 2 |
| W26 | 4 | 0.45 | 2 | 0.50 | 2 |
| W28 | 4 | 0.3 | 2 | 0.50 | 2 |
| W29 | 4 | 0.3 | 2 | 0.40 | 3 |
| W39 | 4 | 0.75 | 2 | 0.80 | 3 |
| SP15 | 4 | 0.3 | 2 | 0.50 | 2 |
| SP21 | 4 | 0.15 | 2 | 0.40 | 2 |
| SP43 | 4 | 0.3 | 2 | 0.30 | 2 |
| SP45 | 4 | 0.3 | 2 | 0.45 | 2 |
| SP47 | 4 | 0.2 | 2 | 0.30 | 2 |
| SP56 | 4 | 0.65 | 2 | 0.90 | 3 |
| W15 | 5 | 0.25 | 2 | 0.25 | 2 |
| W16 | 5 | 0.25 | 2 | 0.50 | 2 |
| W30 | 5 | 0.5 | 2 | 0.25 | 2 |
| W32 | 5 | 0.6 | 2 | 0.75 | 2 |
| SP10 | 5 | 0 | 3 | 0 | 3 |
| SP25 | 5 | 0.25 | 2 | 0.25 | 2 |
| SP30 | 5 | 0.25 | 2 | 0.25 | 2 |
| SP38 | 5 | 0.75 | 2 | 0.75 | 2 |
| SP39 | 5 | 0.25 | 2 | 0.25 | 2 |
| SP48 | 5 | 0.1 | 2 | 0 | 3 |
| W15 | 6 | 0.2 | 1 | 0.20 | 2 |
| W16 | 6 | 0.15 | 2 | 0.40 | 2 |
| W30 | 6 | 0.05 | 1 | 0.10 | 2 |
| W32 | 6 | 0.75 | 2 | 0.80 | 2 |
| SP10 | 6 | 0 | 3 | 0 | 3 |
| SP25 | 6 | 0.05 | 1 | 0.15 | 1 |
| SP30 | 6 | 0.05 | 2 | 0.05 | 2 |
| SP38 | 6 | 0.5 | 2 | 0.75 | 2 |
| SP39 | 6 | 0.05 | 3 | 0.05 | 3 |
| SP48 | 6 | 0.02 | 3 | 0.05 | 3 |
