# Supplementary material for: Using virtual reality and thermal imagery to improve statistical modelling of vulnerable and protected species
Source: PLoS One. 2019 Dec 11;14(12):e0217809. doi: 10.1371/journal.pone.0217809 (PMC6905580; doi:10.1371/journal.pone.0217809)
Supplement: S1 File — Standardized expert-elicitation protocol for practice interviews and expert elicitation. (DOCX) [file pone.0217809.s004.docx]

Supporting information for Leigh et al. “Using virtual reality and thermal imagery to improve statistical modelling of vulnerable and protected species” published by PLoS ONE.

**S1 File. Interview protocol.** Protocol for practice interviews and expert elicitation.

***Practice Interview***

***Introduction.***

This document is part of the multidisciplinary research effort designed to enhance the monitoring of koala presence in southeast Queensland forests. The aim of this part of the project is to obtain information from experts to contribute to this evidence base.

Thank you for agreeing to be an expert in this project. Please note that your answers are not binding in any way. We have asked you to participate because we respect your expertise and we want to learn from you. We will combine your information with the information received from other experts, so that we can build a solid evidence base.

Please note that we can stop the interview at any time. If you experience cybersickness or if you have any other concerns, please advise your interviewer and we can end the interview.

You will be interviewed by a project team member. We will show you a set of images of sites (locations) in Alexander Clarke Park. We will ask you about several things.

Note that there are no correct answers to the following questions. This is not a test. We are asking for your opinion, based on your expertise.

***Instructions.***

Before we start, please summarize your expertise regarding koalas, koala habitats or related topics.

Note that there are no correct answers to the following questions. This is not a test. We are asking for your opinion, based on your expertise.

***Format of questions.***

The questions in today’s interview will require you to think about probabilities in a few different contexts. Some examples about how to think about probability include:

- One way is in terms of a gauge: Empty = 0 ---------- Full = 1
- Or in terms of percentages: 0% = never/impossible ---------- 100% = always/certain
- Or consider going to 10 sites like this one:
  - For the probability of a koala living at the displayed site, you could ask: how many (or what proportion or %) of these 10 sites would have a koala living there?
  - When considering the probability that a koala lives in a site, we also need to take to take into account other factors, such as the time of year. You can include this additional uncertainty by ‘averaging’ over these factors and widening the minimum and maximum probability limits.

Interview questions will be asked in one of the following formats:

Note: These questions ask for the probability/quantity koalas present in a given location – not necessarily for the koala being visible to observers.

**Question 1:** What is the smallest probability of a koala being present in this location?

One way to think of this is to consider going to this location 10 times. What is the minimum number of times you would expect a koala to be present? Another way to think about this is as a percentage: what % of times would a koala be present in this location? 0% = never, 50% = half the times, 100% = every time.

**Question 2:** What is the largest probability of a koala being present in this location?

One way to think about this is to ask: what is the maximum number of times a koala would be present there, out of 10 times?

Note that if a koala is always or never present at a location, then your answer to this question will be the same as your answer to Question 1 above.

**Question 3:** What is the expected probability of a koala being present in this location?

Out of 10 visits to this location, what is the most likely number of times a koala would be present there?

If you gave the same probability for Questions 1 and 2, then you will give the same probability for this question. Otherwise, your answer should be between the two numbers that you gave for Questions 1 and 2. Note that it doesn’t have to be in the middle of those two numbers.

**Question 4:** What is the probability that this is a suitable koala habitat?

This question once again asks for a probability, so recall how probabilities are explained above (e.g. in terms of a gauge or a percentage). This question, however, asks for the probability of a habitat being suitable for a koala, not for the probability of a koala living there.

**Question 5:** How certain are you about your answers?

The degree of certainty with which we answer questions like this depends on many factors. It can reflect the level of expertise about a topic (e.g. your knowledge of koalas and koala habitats), and the variability in the factors that affect the probabilities (e.g. koala behaviour in different environments), etc. Here we want a score of 1, 2, 3 to reflect a general level of certainty that combines all of these factors (1 = not very sure, 2 = quite sure, 3 = very sure). Please note that this question is independent of whether or not you think koalas will be present (e.g., you may believe that there would be lots of koalas but be unsure, or you may believe that there would be few koalas and be very sure).

***Practice questions (given for each of 3 randomly selected practice images).***

Take some time to look around, let us know when you’re ready. Feel free to take the goggles on and off as you please.

Probability of koala presence.

First, we will ask you about the probability of koala presence at this site.

**Q1.** What is the smallest probability of a koala being present in this location?

**Q2.** What is the largest probability of a koala being present in this location?

**Q3.** What is the expected probability of a koala being present in this location?

**Q4.** How certain are you about your answers? (1 = not very sure, 2 = quite sure, 3 = very sure)

Overall suitability

Next, we will ask you about the overall suitability of this site as a koala habitat.

**Q5.** What is the probability that this is a suitable koala habitat?

**Q6.** How certain are you about your answer? (1 = not very sure, 2 = quite sure, 3 = very sure)

This is the end of the practice session. Please raise any questions or concerns with your interviewer before commencing the interview. You are also welcome to ask questions at any time during the interview. Do you have any final questions or concerns?

***Expert Elicitation Interview***

***Introduction.***

This document is part of the multidisciplinary research effort designed to enhance the monitoring of koala presence in southeast Queensland forests. The aim of this part of the project is to obtain information from experts to contribute to this evidence base.

Thank you for agreeing to be an expert in this project. Please note that your answers are not binding in any way. We have asked you to participate because we respect your expertise and we want to learn from you. We will combine your information with the information received from other experts, so that we can build a solid evidence base.

You should have access to a set of images that will be used for this interview. These are different sites in Alexander Clarke Park. We will ask you some questions about each site.

You will be interviewed by a project team member. You should have completed the Practice Exercises. Please confirm this with your interviewer before starting this interview.

Note that there are no correct answers to the following questions. This is not a test. We are asking for your opinion, based on your expertise.

***The questions (given for each of 10 images*** ***selected in a random order from a randomly selected subset).***

Take some time to look around, let us know when you’re ready. Feel free to take the goggles on and off as you please.

When you are ready, we will next ask you about the probability of koala presence at this site:

**Q1.** What is the smallest probability of a koala being present in this location?

**Q2.** What is the largest probability of a koala being present in this location?

**Q3.** What is the expected probability of a koala being present in this location?

**Q4.** How certain are you about your answers? (1 = not very sure, 2 = quite sure, 3 = very sure)

When you are ready, we will ask you about the overall suitability of this site

**Q5.** What is the probability that this is a suitable koala habitat?

**Q6.** How certain are you about your answer? (1 = not very sure, 2 = quite sure, 3 = very sure)

This is the end of the interview. Thank you for your participation.
